# Postpartum Screening and Referral for Pelvic Floor Dysfunction at a United States Military Medical Center

**DOI:** 10.1007/s00192-026-06534-6

**Published:** 2026-03-30

**Authors:** Jenny Sue DeWalt, Tanner Carlock, Kelly Nagy, Halei Wong, Audrey Kreke, Nancy Niemczyk

**Affiliations:** 1 University of Pittsburgh School of Nursing, Pittsburgh, PA USA; 2 Wright-Patterson Air Force Base/Wright State University, 1 Wyoming Street, Dayton, OH 46409 USA

**Keywords:** Incontinence, Pelvic floor dysfunction, Pelvic floor physical therapy, Pelvic organ prolapse, Postpartum, Screening and referral

## Abstract

**Introduction and Hypothesis:**

As more women serve in physically demanding military roles, they are increasingly vulnerable to pelvic floor dysfunction (PFD). Our aim was to improve the pelvic health of postpartum women by creating a standardized screening and referral program for PFD at a US military medical center.

**Methods:**

This quality improvement project utilized a pre-post design, evaluating data from a historical group and an intervention group. The intervention utilized a standardized screener to assess women for PFD at a 2-week postpartum phone call and a 6-week postpartum clinic visit. The primary outcome was referral rate to pelvic floor physical therapy (PFPT). Additional variables assessed included screening compliance, referral utilization, and provider satisfaction.

**Results:**

There were 122 postpartum women in the historical group and 181 in the intervention group. The referral rate nearly doubled when comparing the historical and intervention groups (17.2% vs 33.1%, *p* = 0.002). Referral utilization was also higher in the intervention group (18.2% vs 14.8%, *p* < 0.001). At the time of the 2-week phone call, screening compliance was 96%, compared with 67% at the 6-week visit. A pre-intervention provider survey indicated that most providers screened for PFD without a standardized screening tool. A post-intervention survey indicated that the implemented screening process helped to direct patient conversations, was feasible, and assisted with identifying dysfunction.

**Discussion:**

A universal standardized screening program for PFD in postpartum patients at a US military medical center was significantly associated with increased PFPT referral and utilization. Time was the biggest barrier to screening. Providers were largely supportive and accepting of the screening process.

**Supplementary Information:**

The online version contains supplementary material available at 10.1007/s00192-026-06534-6

## Introduction

Approximately 25% of women in the USA live with pelvic floor dysfunction (PFD), including symptoms such as urinary incontinence, fecal incontinence, and pelvic pain [1]. These symptoms negatively impact women across the lifespan, with pregnancy recognized as a significant risk factor [2, 3]. Large multicenter studies have shown that approximately 49% of pregnant and postpartum women experience at least 1 PFD-related symptom [4]. Although some symptoms are transient and resolve within the 1st year postpartum, a substantial number of women continue to experience persistent issues well beyond the postpartum period [3, 5, 6]. Women serving in the military may face an increased risk of developing PFD owing to the physical demands of duty, including rigorous training, heavy lifting, operating heavy machinery, and serving in combat roles [1]. These challenges can significantly affect a service member’s ability to deploy, hinder their career progression, diminish the overall quality of their military experience, and impact long-term military retention [1].

Pelvic floor physical therapy (PFPT) is considered a first-line conservative intervention for PFD. Numerous studies have demonstrated that both pelvic floor muscle training through home exercise programs and in-person PFPT sessions are effective at reducing symptoms [5, 7–12]. However, women frequently refrain from initiating discussions about PFD owing to the sensitive nature of these conditions, and many health care providers do not routinely screen for them [13]. Although providers in other high-income countries commonly refer patients to PFPT, screening and referral practices among providers in the US remain inconsistent, limiting patient awareness and access to timely treatment [3, 13].

Proactive evaluation and treatment are essential because pelvic weakness may be asymptomatic, and the physical toll of childbirth is cumulative with each delivery. Early referral is also critical, as prompt intervention can prevent progression of PFD, support recovery, and reduce the risk of long-term symptoms. These considerations are particularly relevant for military members, who rapidly resume physically demanding occupational duties. Thus, incorporating structured early postpartum screening may help to identify otherwise unrecognized dysfunction and promote recovery.

The US military does not currently have a standardized PFD screening process for postpartum patients [2]. The US National Defense Authorization Act for the Fiscal Year 2022 mandated the establishment of clinical practice guidelines for pelvic health screening of postpartum military members or military dependents [14]. The regulatory organization for the military health system, the Defense Health Agency (DHA), issued a Practice Recommendation in 2022 for all military health care providers, which promoted pelvic health screening for all women annually and for postpartum women at their first comprehensive postpartum examination [2]. The DHA also recommended the use of a standardized questionnaire, specifically, the Cozean Pelvic Dysfunction Screening Protocol [2]. Following screening, referral to PFPT is recommended for women who screen positive for PFD.

This project was aimed at improving the pelvic health of postpartum women by creating, piloting, and evaluating a standardized screening and referral program for postpartum patients at a large United States Air Force (USAF) medical center. It also sought to bring this institution into compliance with DHA standards and promote a fit military force. It was hypothesized that implementation of a universal PFD screening tool would increase the PFPT referral rate in postpartum women and improve the treatment of PFD. As no nationally standardized postpartum PFD screening model currently exists in the USA, establishing a consistent process within the USAF may offer insights for broader postpartum care. Therefore, this project has relevance not only for meeting DHA standards and supporting military readiness but also for informing efforts to standardize postpartum pelvic health assessment nationally.

## Materials and Methods

### Setting and Participants

The study was conducted at a USAF medical center located within the continental USA. This facility provides comprehensive health care services to military personnel, their dependents, and military retirees. The site offers full-spectrum obstetric and gynecological (OB/GYN) care, including both medical and surgical services, delivered by a multidisciplinary team comprising OB/GYN attending physicians, OB/GYN resident physicians, certified nurse-midwives, nurse practitioners, and subspecialists in urogynecology, reproductive endocrinology and infertility, and gynecological oncology. The medical center also has a full-time physical therapist who specializes in PFPT. When patient needs exceed in-house capabilities, providers may refer patients to off-base specialists to ensure appropriate care. The annual delivery volume for this patient population is approximately 300 births.

Patients in the historical group delivered at this military medical center and received their prenatal and postpartum care at the same facility. Those in the intervention group delivered at a local civilian partner hospital, attended by military OB/GYN providers, but received their prenatal and postpartum care at the aforementioned military medical center. The change in delivery location only occurred because of staffing needs and was not related to the implementation of this screening program.

The historical comparison group was analyzed to obtain pre-intervention data and patient characteristics. Inclusion criteria comprised English-speaking US military medical insurance (TRICARE) beneficiaries who experienced a live birth, delivered either vaginally or via cesarean section, and received prenatal care at the aforementioned military facility. Exclusion criteria consisted of patients who were not TRICARE beneficiaries, those who experienced a fetal demise, or those who did not receive prenatal care at the military facility.

This project was reviewed by both the University of Pittsburgh Human Research Protection Office and the Wright-Patterson Air Force Base Human Protection Office, each of which determined that the work met criteria for exemption as a quality improvement initiative. As part of these determinations, all necessary permissions were obtained to access patient information within the Department of Defense (DoD) electronic medical record (EMR) system, facility delivery logs, and the Referral Management Center database. Only personnel with active credentials and authorized EMR access performed data extraction. All information remained within secure DoD networks and was stored in a password-protected, Health Insurance Portability and Accountability Act (HIPAA)-compliant, DoD-approved database. Only de-identified, aggregated data were used for analysis. Because this project was classified as a quality improvement initiative and not human subject research, informed consent was not required, and no participants were asked to sign an informed consent form. Screening and referral processes were conducted as part of routine postpartum clinical care.

### Intervention

This quality improvement project employed a pre–post design. Historical patient data were extracted from a facility delivery log, whereas intervention group patients were identified and tracked through the EMR. Relevant demographic and screening information was de-identified and transferred to the aforementioned secure database. Data on PFPT referral utilization were obtained from the on-base Referral Management Center, which monitors referral orders, acceptance, and utilization. For the historical group, 9 patients could not be located in the EMR and were excluded. A sensitivity analysis assuming that all 9 had received PFPT referrals showed minimal impact on overall referral rates, indicating that exclusion did not significantly alter study results.

The screening tool utilized for this project was the Cozean Pelvic Floor Dysfunction Screening Protocol. It was chosen largely on the basis of DHA endorsement in the aforementioned DHA Practice Recommendation, in addition to its reported sensitivity and ease of use [2]. This tool was developed by a physical therapist who specializes in PFPT and was introduced in 2017 at the International Pelvic Pain Society. It has shown promising preliminary validity, demonstrating a sensitivity of 91% [15]. More extensive validation is ongoing, and further studies are needed to fully establish the tool’s reliability and generalizability across diverse postpartum populations. This one-page tool asks ten questions regarding urinary incontinence, anal incontinence, pelvic pain, pelvic organ prolapse, and pain during intercourse. A score of ≥ 3 warrants a PFPT referral [2, 16].

Per the project protocol, patients were screened for PFD at two designated postpartum time points: 2 weeks and 6 weeks after delivery. The initial screening was conducted via telephone at 2 weeks postpartum, and the second was conducted in-person at 6 weeks postpartum.

These appointment intervals reflect both guideline-supported and standard clinical practice [17]. The 2-week time point was chosen in accordance with American College of Obstetricians and Gynecologists (ACOG) Committee Opinion 736 on optimizing postpartum care, which recommends early postpartum contact within the first 3 weeks after delivery [17]. We incorporated PFD screening into this phone contact already being conducted at the military facility. The 6-week visit corresponded to the routine comprehensive postpartum evaluation, typically performed at 6 weeks postpartum. This complies with ACOG recommendations that comprehensive postpartum follow-up be completed within 12 weeks of delivery [17].

During the 2-week phone call, the Cozean screening tool was verbally reviewed with patients, and referrals to PFPT were placed when clinically indicated. That is, when patients screened positive on the Cozean Pelvic Floor Dysfunction Screening Protocol, defined as endorsing three or more items on the ten-question screener, they were referred to PFPT.

At the 6-week postpartum office visit, patients completed printed copies of the Cozean screener, which were subsequently reviewed with their provider. As with the 2-week telephone screening, referrals to PFPT were placed when Cozean screening results met the threshold for a positive screen, defined as endorsing three or more items on the ten-question screener. This determination was made by the patient’s treating provider based on the patient’s responses on the Cozean tool, and no additional clinical judgment beyond the standardized cutoff was used to trigger a referral.

**Fig. 1:**
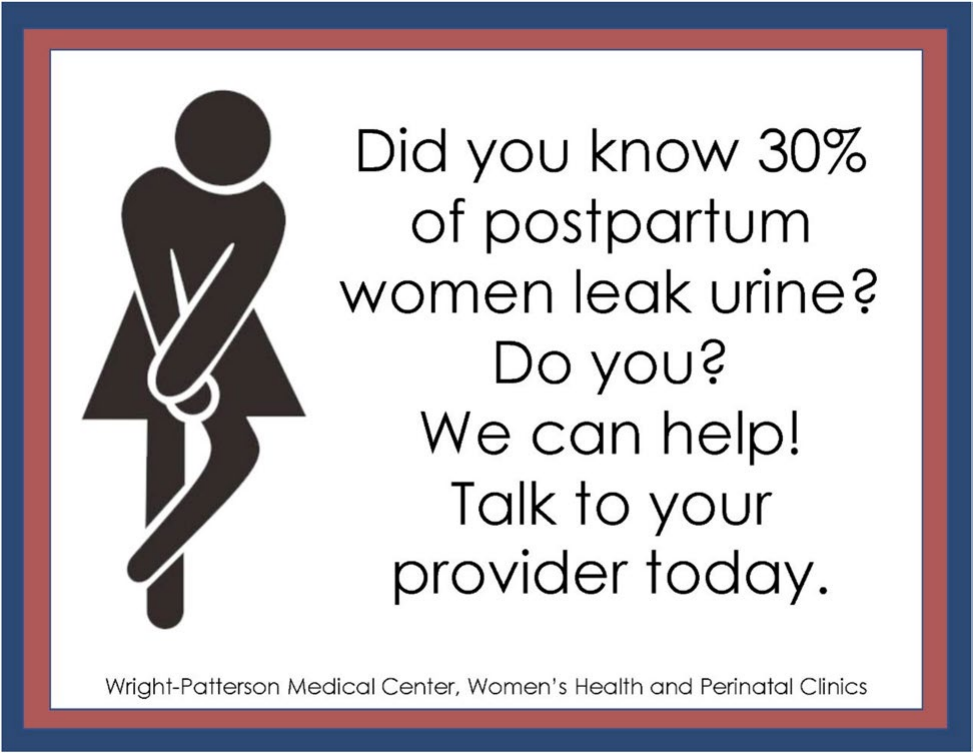
Clinic educational signage

To enhance patient engagement and awareness, simple educational signage regarding pelvic health was also displayed throughout the clinic (Fig. 1). This intervention was based on prior evidence demonstrating a statistically significant increase in incontinence screening rates when patient education signage was utilized [18].

Prior to implementation, the project team conducted two targeted educational sessions reviewing the purpose and protocol for the project, which providers and clinic support staff attended. To streamline workflow and promote consistent documentation, an EMR “dot phrase” was developed and disseminated. This functionality allowed providers to enter a short command into the patient’s chart, which automatically populated the Cozean screening tool for efficient and standardized documentation [19].

A pre-intervention survey was also administered to all providers via online survey software (Qualtrics), querying their screening habits regarding PFD in postpartum patients (Supplementary File 1). The project spanned from October 2023 to May 2024. Patient screening was verified by reviewing a database used to track completion of the 2-week phone call, EMR data from the 6-week visits, and referral data, all done periodically throughout the project’s duration. At the project’s close, all providers completed an additional online questionnaire querying their satisfaction with the screening and referral process (Supplementary File 1).

### Assessment

The primary outcomes of interest were provider screening compliance and the number of PFPT referrals generated. Secondary outcomes included referral utilization, defined as the number of patients who attended at least one PFPT session, and provider satisfaction with the PFD screening and referral process.

Phone screening compliance was calculated as the percentage of study-eligible postpartum patients screened via telephone relative to the total number of these patients called. Similarly, office visit screening compliance was measured as the percentage of study-eligible postpartum patients screened during in-person visits relative to the total number of these patients seen. Referral rates and utilization were reported using both frequencies and percentages.

Referral utilization was evaluated using two metrics: the percentage of the entire cohort who attended at least one PFPT appointment, and the percentage of patients who attended at least one appointment among those who received a referral. Because the historical cohort was likely under-screened for PFD, the primary metric of interest was the proportion of the total cohort who ultimately accessed PFPT, as this measure more accurately reflects connection to therapy between groups.

Statistical analysis was performed using IBM SPSS Statistics version 29.0. For demographic comparison, Chi-squared test was used for categorical variables, and the Mann–Whitney *U* test was used for non-normally distributed continuous variables. For the referral rate, the Chi-squared test was used. For screening compliance, percentages were used. Provider feedback was measured using frequencies and a five-point Likert scale.

A pre-intervention sample-size calculation was performed based on an anticipated increase in PFPT referral rates from a baseline of 26% to 49% post-intervention, with 90% power and a two-tailed alpha of 0.05 [4]. This yielded a required sample size of 91 patients per group (182 in total). Based on historical delivery rates, the project was initially projected to span 6 months to obtain the required number of patients. Accordingly, the historical cohort was drawn from a 6-month period prior to implementation, yielding a total of 122 patients.

During implementation, the intervention period was extended beyond 6 months to 8 months to ensure that at least 91 patients completed the screening process. Extension was necessary owing to a lower-than-expected contact rate at the 2-week postpartum telephone screening, as many patients could not be reached despite multiple attempts (*N* = 71, 40%).

## Results

The sample included 122 postpartum women in the historical group and 181 in the intervention group. Comparison of groups at baseline revealed that groups were similar in ethnicity, military status, mode of birth, operative birth rate, and parity (*p* values all > 0.05; Table 1). Of note, 40.2% of the historical group were military members, whereas 35.4% of the intervention group were military members. Military members included people who were on active duty, active guard, or reserve, or on inactive guard or reserve. The remaining patients were dependents, retirees, or other TRICARE beneficiaries.

**Table 1:** Demographic and clinical information

	Historical control group, *N* = 122	Intervention group, *N* = 181	*p* value
Age: median (interquartile range)	29 (26–32)	31 (27–34)	0.02
Ethnicity: frequency (%)			
Hispanic or Latino	13 (10.7)	18 (10.2)	0.89
Race: frequency (%)			
Black or African American	15 (12.3)	8 (4.6)	0.01
White	87 (71.3)	149 (84.7)	
Other	20 (16.4)	19 (10.8)	
Military member status: frequency (%)			
Military member	49 (40.2)	64 (35.4)	0.39
Weeks postpartum at referral: mean (standard deviation)	5.76 (2.2)	4.47 (2.3)	0.02
Mode of birth: frequency (%)			
Vaginal birth	97 (79.5)	137 (75.7)	0.44
Cesarean birth	25 (20.5)	44 (24.3)	
Parity: frequency (%)			
Primiparous	45 (36.9)	68 (37.6)	0.90
Multiparous	77 (63.1)	113 (62.4)	

Age, race, and weeks postpartum all showed statistically significant differences between groups at baseline. The difference in mean age was due to outliers in both groups—1 patient aged 51 years in the intervention group, and a combined 22 patients from both groups were in their 40 s (6 in the historical group, 16 in the intervention group). The median age and interquartile range were comparable between groups (29 years old [26–32] vs 31 years old [27–34]).

The historical group was more racially diverse (*p* = 0.01), with a higher percentage of women identifying as Black/African American (12.3% vs 4.6%) and a greater proportion belonging to racial groups with small sample sizes (16.4% vs 10.8%), including American Indian or Alaska Native, Asian, Native Hawaiian or Pacific Islander, and individuals who selected a written racial identity. These racial groups were combined into a single analytical category for statistical purposes.

Patients received PFPT referrals earlier in the intervention group than in the historical group (4.5 weeks vs 5.8 weeks). Obstetric anal sphincter injuries (OASIS), defined as all documented third- and fourth-degree vaginal lacerations in the labor and delivery record, were similar in the two groups (historical group 0%, intervention group 2.8%, *p* = 0.084).

The primary outcome was PFPT referral rate (Table 2), which nearly doubled with the intervention group compared with the historical group (33.1% vs 17.2%, *p* = 0.002). Sensitivity analysis results (regarding historical group patients who were not located in the EMR) indicated that if all 9 patients had received a referral, the change in referral rate between groups would be marginally statistically significant (*p* = 0.049). Of those who received a referral in the intervention group, 45% of patients received it after the 2-week phone call, and 55% of patients received it after their 6-week in-person appointment.

**Table 2:** Results: pelvic floor physical therapy (PFPT) referral and utilization rates

	Pre-intervention, *n* (%)	Post-intervention, *n* (%)	*p* value
PFPT referral rates			
Referral placed within 13 weeks of delivery	21 (17.2)	60 (33.1)	0.002
PFPT referral utilization rates			
Attended at least one PFPT appointment	18 (14.8)	33 (18.2)	<0.001

Secondary outcomes analyzed included referral utilization, screening compliance, and provider satisfaction. Referral utilization was higher in the intervention group (18.2% vs 14.8%, *p* < 0.001; Table 2). At the conclusion of the study period, 110 patients in the intervention group had screening completed and documented. At the 2-week phone call, screening compliance was 96.4%, compared with 67.3% at the 6-week visit. A total of 71 patients (40%) could not be reached for the 2-week call and therefore, were not screened at that time. Of the patients reached, only 4 declined participation. The intervention group had 88.5% attendance at the 6-week postpartum visit, of which 67.3% had PFD screening completed and documented.

The pre-intervention provider survey responses indicated that most were screening for PFD without using a standardized screening tool. The 26 providers who responded to this survey indicated that time and lack of training were the biggest barriers to screening. The post-intervention survey had a lower response, with only 10 providers participating. Three providers commented that the screening process increased appointment time, whereas 9 agreed that the screening process directed patient conversations, was helpful, was feasible, and improved patient quality of life. All agreed that the tool helped to identify patients with PFD. The majority of respondents (90%) were satisfied with the screening process. Respondents were split on whether they would continue using the screening tool (5 “yes,” 5 “no”) and recommend it to others (6 “yes,” 4 “no”).

## Discussion

Implementation of a standardized PFD screening and referral program for postpartum patients at a USAF medical center resulted in a doubling of PFPT referral rates, a statistically significant improvement. This demonstrates the effectiveness of systematic screening and suggests that screening at both 2- and 6-week postpartum intervals facilitates earlier identification of PFD and improves access to treatment.

Additional key findings were that patients in the intervention group received PFPT referrals earlier (a mean of 4.5 weeks postpartum) compared with the historical group (a mean of 5.8 weeks postpartum). This difference is attributed to the 2-week phone call screening, which facilitated early detection of symptoms and timely intervention. Early postpartum follow-up, as recommended by ACOG, and subsequent PFPT referral, align with recommendations that emphasize the benefits of prompt PFPT. This pattern avoids delaying intervention prior to resumption of normal activity (e.g., exercise, intercourse) and mitigates long-term dysfunction.

Referral utilization improved in the intervention group compared with the historical group, representing a statistically significant increase. This suggests that standardized screening might not only identify more patients with PFD but also meaningfully enhance access to treatment. Although this represents a clinically important improvement, the increase was smaller than anticipated. Future research should explore barriers to patient engagement with PFPT.

Although screening occurred at the early 2-week postpartum phone call, formal education and therapy often began later (mean 4.5 weeks), which may have led to delayed patient engagement. Future studies could evaluate earlier education and tracking of quality-of-life/functional outcomes at multiple time points, with or without supervised PFPT.

Screening compliance by providers varied between time points. Although compliance during the 2-week telephone encounters—conducted primarily by resident physicians within a structured workflow—was consistently high, adherence at the 6-week postpartum visit was more variable across provider types. Lower screening adherence at 6 weeks likely reflects competing clinical demands during the comprehensive postpartum visit, variable familiarity with the screening protocol, and differing levels of provider engagement. These challenges notwithstanding, the findings suggest that remote screening is both feasible and effective for early postpartum assessment. Additionally, some providers documented screening results inconsistently, using either score-based entries or general narrative notes. Despite the implementation of a standardized EMR “dot phrase,” adherence varied. These findings underscore the need for sustained provider education and workflow reinforcement. With continued familiarity and institutional support, integration of this screening process into routine practice would likely improve.

Provider feedback was overall positive. Pre-intervention survey responses indicated that most providers screened for PFD informally, citing time constraints and lack of training as major barriers to standardized screening. Post-intervention responses showed that providers felt that the screening tool facilitated patient conversations and improved care. However, despite perceived benefit, provider buy-in remained limited, with only half reporting willingness to continue using the tool or recommend it to others. This lack of buy-in and the decrease in survey responses may reflect differences in workflow demands, variable provider engagement, or competing clinical responsibilities. These same factors likely contributed to variability in screening compliance despite training efforts. Future implementation efforts may benefit from emphasizing patient-centered quality-of-life outcomes, demonstrating workflow efficiency, and incorporating targeted provider training to promote sustained engagement.

Our findings are consistent with prior literature demonstrating that PFD is common in pregnant and postpartum populations, yet frequently under-identified and treated. Multiple studies have reported a high prevalence of postpartum pelvic floor symptoms, including urinary incontinence and pelvic pain, while noting variability in screening and referral practices [1, 4, 5]. Similar to prior work showing that structured approaches increase discussion and recognition of pelvic floor symptoms, the implementation of a standardized screening protocol in this project was associated with a significant increase in PFPT referrals compared with historical care [13, 18]. Additionally, earlier screening aligns with existing evidence supporting the benefits of prompt identification and initiation of PFPT in the postpartum period [3, 8–11]. Collectively, these findings reinforce prior recommendations advocating for systematic pelvic floor assessment as part of postpartum care and suggest that structured screening might help to bridge the gap between the prevalence and treatment of PFD observed in earlier studies [2, 6].

Strengths of this quality improvement project include its collaborative, systematic approach, large sample size, and integration of a standardized screening tool (Cozean Pelvic Dysfunction Screening Protocol). The project’s structured early screening at two distinct time points allowed for a comprehensive assessment of postpartum PFD with the opportunity for early referral. Additionally, provider education and the implementation of clinic signage likely contributed to increased patient awareness and willingness to discuss PFD.

Limitations include the reliance on self-reported screening compliance and variability in provider documentation, which may have led to an underestimation of true screening rates. These findings should be interpreted cautiously, given the low post-intervention survey response (*n* = 10). Voluntary participation introduces the possibility of response bias, as providers with more favorable views of the screening process may have been more likely to respond. As such, these results may not be representative of all providers and should be considered exploratory.

Additionally, as this study was conducted at a single military medical center, the findings may not be fully generalizable to other military or civilian health care settings. Future efforts should explore system-wide implementation, including EMR integration, standardized provider training, and institutional support to sustain and scale PFD screening across multiple treatment facilities. Future research should review this implementation and investigate long-term outcomes following PFPT referral and treatment.

Women in the historical and intervention groups gave birth in different clinical settings, either at an on-base military facility or at a partner civilian hospital respectively. Importantly, both groups still received all prenatal and postpartum care at the same military facility. Although the change in delivery site was unrelated to the project, differences in institutional workflows, staffing, and documentation practices may contribute to variability in care. This represents a potential source of bias, which should be considered when interpreting findings.

Additionally, for statistical analysis, racial groups with small sample sizes were combined into a single analytic category. This was done solely to ensure statistical validity rather than to minimize the importance of these identities. However, this approach results in a heterogeneous composite group and may obscure meaningful differences between racial populations. Therefore, these findings should be interpreted cautiously, and future studies with larger and more diverse samples are needed to better evaluate potential racial and ethnic differences in postpartum PFD.

Last, this study utilized the Cozean Pelvic Floor Dysfunction Screening Protocol, as recommended by our governing body, DHA, to identify postpartum PFD. Although the tool has demonstrated promising preliminary sensitivity, it has not been fully validated in postpartum populations. As a result, the findings may be subject to measurement bias, and the reported prevalence of PFD and referral rates should be interpreted with caution. Future studies are needed to further validate this screening instrument and assess its reliability and generalizability in diverse postpartum populations. These factors limit the interpretability of the findings and should be considered when drawing conclusions from this quality improvement initiative.

In summary, a standardized screening program for PFD in postpartum patients was significantly associated with an increased PFPT referral and utilization rate at this US military medical center. Time was identified as the biggest barrier to screening, and providers were supportive and accepting of the screening process. Although challenges remain in provider adherence, documentation practices, and referral utilization, the findings support the continued development of structured screening programs to enhance postpartum pelvic health. Future projects should focus on integrating screening tools within the EMR, addressing provider barriers, and investigating patient-level factors affecting treatment follow-through to maximize the impact of recommended interventions.

## Supplementary Information

Below is the link to the electronic supplementary material.

Supplementary file1 (DOCX 22.6 KB)

Supplementary file2 (PDF 798 KB)

## Data Availability

The data that support the findings of this study are available from the corresponding author upon reasonable request.
