# Supplementary material for: Postpartum Screening and Referral for Pelvic Floor Dysfunction at a United States Military Medical Center
Source: Int Urogynecol J. 2026 Mar 30;37(8):2597–604. doi: 10.1007/s00192-026-06534-6 (PMC13486064; doi:10.1007/s00192-026-06534-6)
Supplement: Supplementary file 1 [file 192_2026_6534_MOESM2_ESM.pdf]

Defense Health Agency
Deputy Assistant Director, Medical Affairs

Women and Infant Clinical Community

DHA Practice Recommendation:
Pelvic Health Evaluation, Treatment, and Referral for Women
*Edition 1**:** Version 1*
Date: September 28, 2022

Defense Health Agency
Falls Church, Virginia

Pelvic Health Evaluation, Treatment, and Referral for Women

Edition 1: Version 1

2022

Release Authority: Dr. Paul R. Cordts, Deputy Assistant Director - Medical Affairs, Defense Health Agency

Document is unclassified / for official use only.

Editors: See authors and affiliations

Support From: Women and Infant Clinical Community

To Cite: Weir et. al (2022). *Pelvic Health Evaluation, Treatment, and Referral for Women.*

DHA Practice Recommendation: Overview and Disclaimer

DHA Practice Recommendations (PRs) are developed by experts utilizing the best information available at the time of publication. In some instances, some recommendations are expert opinion provided to users in the absence of definitive, well-designed and executed randomized control trials. DHA's PRs provide the field with an authoritative source of carefully synthesized clinical information. They are intended to assist clinical care teams with real-time decision making based on best available evidence.

While the DHA sponsors this PR, its endorsement of the findings and recommendations are limited to validation of the expert opinion and compiled evidence of the sponsoring Subject Matter Expert (SME) body. This PR should be used to augment the practitioner's best clinical judgment. It may not account for local or structural conditions (i.e., resourcing, staffing, equipment, or Health Protection Conditions) impacting clinical decision making in the field by the practitioner.

DHA PRs are separate and distinct from jointly developed Department of Veterans Affairs (VA) / DoD Clinical Practice Guidelines that are the product of rigorous, systematic literature review and synthesis. In contrast, DHA PRs provide the DHA practitioner with a synopsis of relevant clinical evidence tailored to the military medicine setting and TRICARE beneficiary population.

DHA PRs provide standardized evidence-informed guidelines that DHA practitioners should refer to when addressing patients with specific clinical conditions. Clinical practitioners must be mindful of the emergence of supervening clinical evidence published in the academic press not yet incorporated into the guideline.

This guideline is not intended to define a standard of care and should not be construed as such, nor should it be interpreted as prescribing an exclusive course of management for said condition or disease process. Variations in practice will inevitably and appropriately occur when clinicians consider the needs of individual patients, available resources, and limitations unique to an institution or type of practice. Every healthcare professional making use of this guideline is responsible for evaluating the appropriateness of applying it in the setting of any particular clinical situation.

This guideline is not intended to represent TRICARE policy. Further, inclusion of recommendations for specific testing and/or therapeutic interventions within this guide does not guarantee coverage of Private Sector Care. Additional information on current TRICARE benefits may be found at www.tricare.mil or by contacting the regional TRICARE Managed Care Support Contractor.

Table of Contents

DHA Practice Recommendation: Overview and Disclaimer iii
Pelvic Health Evaluation, Treatment, and Referral for Women: Edition 1: Version 1 1
Setting 1
Purpose 1
Diagnosis 1
Clinical Management 2
References 5
Authors and Affiliations 5
Statement of Authorship 5
Potential Conflicts of Interest 6
External Peer Review 6
Approved By 6

Pelvic Health Evaluation, Treatment, and Referral for Women: Edition 1: Version 1

WICC 2022-01	
September 12, 2022	Date of Expiry – N/A
Previous Document Number – N/A	Supersede Date – N/A

Setting

All military Medical Treatment Facilities (MTFs) and outpatient clinicians providing care to women in the Defense Health Agency (DHA).

Purpose

This DHA PR provides DHA practitioners who care for women standardized recommendations on: 1) evidenced-informed guidelines for using the Cozean Pelvic Dysfunction Screening Protocol (Figure 1)[1], for pelvic health evaluation; 2) recommended intervals for pelvic health screening; and 3) considerations for healthcare providers on treatment modalities and/or when a referral process for pelvic floor health physical therapy should be initiated. This DHA PR also includes guidance for the appropriate use of telehealth services for initial evaluation and history of symptoms (using the Cozean Pelvic Dysfunction Screening Protocol (Figure 1)) and for follow-up, in order to help improve readiness and daily living.

The "pelvic floor" is the group of muscles that form a sling or hammock across the floor of the pelvis.[2] Together with the surrounding tissues, these muscles support the pelvic organs (i.e., bladder, urethra, intestines, and rectum) in its anatomic place so they can function correctly. A woman's pelvic organs also include the uterus, cervix, and vagina.[3]

A pelvic floor disorder (PFD) occurs when the pelvic muscles are not functioning properly, often due to weakened or damaged pelvic floor muscles or due to chronic spasm of the pelvic floor muscles. Many cases of PFD are underreported and/or underdiagnosed, due to the complexities of symptoms related to the pelvic region, and misconception by many medical professionals who believe the signs and symptoms of PFD are a normal part of aging.[4] Additionally, some symptoms of PFD, such as chronic pelvic or low back pain, may not be recognized as a clinical spectrum of PFDs. Compounding these challenges, PFD conditions can contribute to chronic pain syndromes, social isolation, reduced mobility, a higher fall risk, depressive symptoms, distress, and reduced quality of life as well as impacting military Service member medical readiness.[5,6] PFD does not have to be a lifelong condition. With timely assessment, evaluation, and treatment, the symptoms of PFD may be reduced or eliminated altogether.

Diagnosis

PFD presents with a variety of symptoms, many times not directly attributed to the pelvic area such as back pain, fatigue, or general 'fullness.' Causes of PFD are multi-factorial and include age-related atrophy, nerve and tissue injury secondary to childbearing/childbirth, repetitive stress (repetitive heavy lifting or chronic chough or constipation), and genetic factors. PFDs can also develop into mental health disorders and physical or psychological trauma.

The most common types of PFDs include pelvic organ prolapse, bladder and bowel leakage, lower urinary tract symptoms, and chronic pelvic pain. The female pelvic anatomy predisposes women to higher rates of PFDs vs. men. Men can experience nearly all of the same PFDs as women, but they are less common. Specific female examples include dyspareunia, and PFDs that interfere with a woman's reproductive health. Women may also have symptoms such as chronic pelvic pain or pain during sex.[7] The definitive causes of PFD are unknown, but risk factors include traumatic injuries to the pelvic area (like a car accident), pregnancy, chronic strain on the pelvic musculature (i.e., repetitive lifting or jumping, chronic cough, constipation, and potentially gravitational force stress on the pelvic floor) leading to poor muscle coordination, pelvic surgery, being overweight, and/or advancing age.

Often, PFD remains underdiagnosed with many women suffering with their symptoms for many years before seeking evaluation due to embarrassment or the misconception that there is nothing that can be done to help their symptoms. Additionally, not all PFD can be completely quantified by objective tests or imaging, and healthcare providers must rely on patient reported symptoms.

In the absence of clearly defined clinical management guidelines, this DHA PR is designed to provide MTF clinicians (i.e., Direct Care) evidenced-based strategies for pelvic health evaluation and associated treatment for women in the DHA. Using the guidance provided, MTFs should adapt these guidelines to implement their own Standard Operating Procedures (SOPs) for safe and effective pelvic health evaluation and treatment. MTF SOPs should include at minimum the following processes: staff training on proper screening for PFD, referral criteria for treatment, and validation of consistent utilization of standardized processes, using the Cozean Pelvic Dysfunction Screening Protocol (Figure 1). Final judgment and clinical discretion regarding pelvic health evaluation and referral remains with the medical provider/team.

Clinical Management

The following clinical management procedures for pelvic floor evaluation and referral should be used:

Asymptomatic patients:

- Screening questions directed at identifying pelvic health problems (e.g., assess sexual, urinary, and bowel function) should be included at least annually during comprehensive health evaluations. This may be completed in primary care (e.g., Primary Care Manager visit) or specialty care (e.g., women's health visit).
  - For most patients, screening should consist of use of the Cozean Pelvic Dysfunction Screening Protocol (Figure 1).
    - Patients responding 'yes' on three or more questions are likely experiencing PFD, and should receive further evaluation and/or treatment.
    - Further evaluation and treatment may include the following, either alone or in combination: physical therapy (pelvic floor), chronic pain management, and/or behavioral health.
    - When/where appropriate, telehealth may also be an effective tool for initial evaluation, history of symptoms, follow-up, and in some cases treatment as a way to positively impact readiness/daily living.
- Pelvic exams should not be routinely conducted in asymptomatic patients.

   - The American College of Obstetricians and Gynecologists (ACOG) recommends women have pelvic exams only when they have symptoms or have a medical history that requires an exam.[8] Ultimate discretion for the performance of a pelvic exam would lie with the patient and the medical provider/team.

Symptomatic patients or patients with a history of gynecological or genitourinary conditions:

- A pelvic evaluation should be performed for patients experiencing symptoms for and/or with a history of abnormalities that suggest a pelvic disorder. Symptoms may include, but are not limited to, abnormal bleeding, abnormal vaginal discharge, pelvic pain, incontinence of bowel or bladder, prolapse of pelvic organs, and/or pain during sex.
   - Additional tests or procedures may be needed to help diagnose the cause of the symptoms. These may be pursued by either primary care or specialty care providers.
- Depending on a provider's assessment and specific symptoms/diagnosis(es), treatment options may include medications (e.g., non-opioid analgesics), lifestyle modifications (e.g., diet, exercise), surgery, physical therapy, or counseling, in combination or alone.
   - First line treatment often includes referral to specialty care. Specifically, and most commonly, patients diagnosed with PFD should be referred to pelvic floor physical therapy and/or to specialty care with a women's health provider.
   - The criteria for referring to pelvic floor physical therapy is any symptom of PFD to include urinary urgency or frequency, urinary incontinence, dysfunctional voiding, pelvic pain, constipation, etc. Most PFD can benefit from pelvic floor physical therapy. Physical therapy may not be the only treatment needed, but may be a good first line treatment for the vast majority of women. Many women who respond to physical therapy may not require additional treatment.
- Additional referrals may include pain management, occupational therapy, and/or behavioral health.
- When/where appropriate, telehealth may also be an effective tool for further evaluation and treatment.

Pregnant and postpartum patients:

- Pregnancy and postpartum healing should not be reasons to defer consultations or further evaluation in patients with signs or symptoms of PFD.
- Pelvic health screening should be done at the comprehensive postpartum visit (six to twelve weeks postpartum), with the Cozean Pelvic Dysfunction Screening Protocol (Figure 1). Further screening should be done by the patient's healthcare provider (i.e., primary care or women's health provider) evaluating the patient at four, nine, and twelve months postpartum and/or if symptomatic.
- Healthcare providers should incorporate pelvic health education during pregnancy to increase awareness of common PFDs during pregnancy and postpartum, and help patients advocate for themselves if they have symptoms. Prenatal pelvic health awareness and pelvic floor muscle training may also help mitigate pelvic floor symptoms during pregnancy and postpartum.

Summary

PFDs are likely underdiagnosed with a wide spectrum of clinical manifestations. PFD assessment and management is complex and may likely require a multi-disciplinary team including, but not limited to: primary care, women's health, physical therapy, behavioral health, neuromusculoskeletal, and others,

depending on a patients' specific diagnosis. Early recognition of PFD symptoms followed by appropriate diagnosis and treatment is particularly important for maintaining readiness, minimizing time away from duty, and retention of Active Duty Service Members.

Figure 1: Cozean Pelvic Dysfunction Screening Protocol[1]

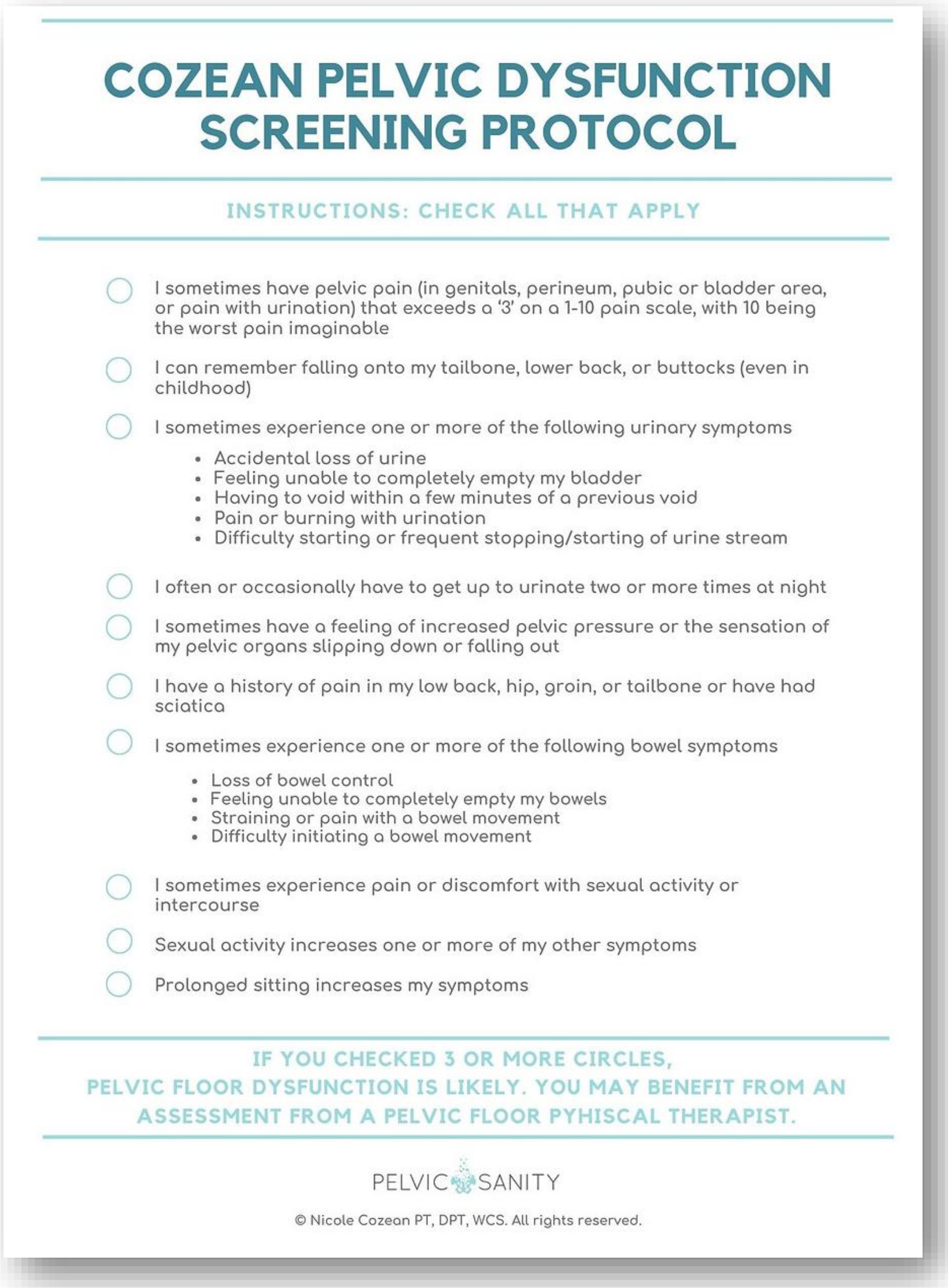

COZEAN PELVIC DYSFUNCTION SCREENING PROTOCOL

INSTRUCTIONS: CHECK ALL THAT APPLY

- ◯ I sometimes have pelvic pain (in genitals, perineum, pubic or bladder area, or pain with urination) that exceeds a '3' on a 1-10 pain scale, with 10 being the worst pain imaginable
- ◯ I can remember falling onto my tailbone, lower back, or buttocks (even in childhood)
- ◯ I sometimes experience one or more of the following urinary symptoms
  - Accidental loss of urine
  - Feeling unable to completely empty my bladder
  - Having to void within a few minutes of a previous void
  - Pain or burning with urination
  - Difficulty starting or frequent stopping/starting of urine stream
- ◯ I often or occasionally have to get up to urinate two or more times at night
- ◯ I sometimes have a feeling of increased pelvic pressure or the sensation of my pelvic organs slipping down or falling out
- ◯ I have a history of pain in my low back, hip, groin, or tailbone or have had sciatica
- ◯ I sometimes experience one or more of the following bowel symptoms
  - Loss of bowel control
  - Feeling unable to completely empty my bowels
  - Straining or pain with a bowel movement
  - Difficulty initiating a bowel movement
- ◯ I sometimes experience pain or discomfort with sexual activity or intercourse
- ◯ Sexual activity increases one or more of my other symptoms
- ◯ Prolonged sitting increases my symptoms

IF YOU CHECKED 3 OR MORE CIRCLES,
PELVIC FLOOR DYSFUNCTION IS LIKELY. YOU MAY BENEFIT FROM AN ASSESSMENT FROM A PELVIC FLOOR PYHISCAL THERAPIST.

PELVIC SANITY

© Nicole Cozean PT, DPT, WCS. All rights reserved.

References

1. Pelvic Sanity. Cozean Pelvic Dysfunction Screening Protocol. Retrieved September 09, 2022, from https://www.pelvisanity.com/post/screening
2. United States Department of Health and Human Services, National Institutes of Health. *Pelvic Pain*. Retrieved May 25, 2022, from https://www.nichd.nih.gov/health/topics/pelvicpain
3. United States Department of Health and Human Services, National Institutes of Health. About Pelvic Floor Disorders. Retrieved May 25, 2022, from: https://www.nichd.nih.gov/health/topics/pelvicfloor/conditioninfo
4. Schüssler-Fiorenza Rose SM, Gangnon RE, Chewning B, et al. Increasing discussion rates of incontinence in primary care: a randomized controlled trial. *J Women's Health*. 2015; 24(11): 940–9. 10.1089/jwh.2015.5230
5. Parvaiz M. Improving the care pathway for women with incontinence. *Women's Health*. 2015;11(2):105–8. 10.2217/WHE.14.83. Medline:25776284
6. Pizarro-Berdichevsky J, Hitschfeld MJ, Pattillo A, et al. Association between pelvic floor disorder symptoms and QoL scores with depressive symptoms among pelvic organ prolapse patients. *Aust N Z J Obstet Gyn*. 2016;56(4):391–7. 10.1111/ajo.12467. Medline:27135639
7. Cleveland Clinic. Pelvic Floor Dysfunction. Retrieved May 26, 2022, from: https://my.clevelandclinic.org/health/diseases/14459-pelvic-floor-dysfunction
8. ACOG. Pelvic Exams: Frequently Asked Questions. Retrieved May 25, 2022, from https://www.acog.org/womens-health/faqs/pelvic-exams

Authors and Affiliations

Col Larissa Weir, MD, FACOG
DHA WICC Air Force Representative
AF/SG Chief Women's Health Consultant, Air Force Medical Readiness Agency

CDR Angela DiCarlo-Meacham, MD, FACOG
WICC Navy Representative
Female Pelvic Medicine and Reconstructive Surgery, Naval Medical Center San Diego

Ms. Janet Papazis, PT, DPT
DHA Neuromusculoskeletal Clinical Community Program Manager
Clinical Support Division, Defense Health Agency

Ms. Theresa Hart, MS, RN, NCC-E
DHA WICC Program Manager
Clinical Support Division, Defense Health Agency

Statement of Authorship

All listed authors conceptualized the document, reviewed, and revised the document critically for important intellectual content, approved the final document submitted, and agreed to be accountable for all work aspects. Col Larissa Weir will also ensure questions related to the accuracy or integrity of any part of the work is appropriately investigated and resolved.

Potential Conflicts of Interest

The Authors declare no conflicts of interest.

External Peer-Review

N/A

Approved By

Paul R. Cordts

8 October 2022

PAUL R. CORDTS, MD
Deputy Assistant Director - Medical Affairs
Defense Health Agency

Date of Signature
