# Supplementary material for: Postpartum Screening and Referral for Pelvic Floor Dysfunction at a United States Military Medical Center
Source: Int Urogynecol J. 2026 Mar 30;37(8):2597–604. doi: 10.1007/s00192-026-06534-6 (PMC13486064; doi:10.1007/s00192-026-06534-6)
Supplement: Supplementary file 2 [file 192_2026_6534_MOESM1_ESM.docx]

Appendix 1: Provider Questionnaires

**Pre-Intervention Survey: Provider PFD Screening Habits Questionnaire**

Email Address (fill in blank)

Provider Designation (drop-down): OB/GYN, CNM, NP, Resident (PGY-1, PGY-2, PGY-3, PGY-4)

Years OB experience: 0-2, 2-5, 5-10, 10-15, 15-20, 20+

1. How do you currently screen patients for Pelvic Floor Disorders such as urinary incontinence, anal incontinence, pelvic organ prolapse, or painful intercourse? (Select 1 or blank space to fill out): Screening tool, Questions I’ve created and incorporated into my assessment, Wait for patient to bring up concerns, Other, I don’t.
2. If you use a screening tool to assess for postpartum Pelvic Floor Disorders, which one do you use? (Select 1 or blank space to fill out: “My own personalized questions”, Pelvic Floor Distress Inventory-20 (PFDI-20), Pelvic Floor Impact Questionnaire-7 (PFIQ-7), Modified Pelvic Floor Questionnaire (mPFQ), International Consultation on Incontinence Questionnaire—Urinary Incontinence Short Form (ICIQ-UI SF), International Consultation on Incontinence Questionnaire—Vaginal Symptoms (ICIQ-VS), Oswestry Disability Index, Blank space to fill out, or N/A)
3. If you do not routinely screen for Pelvic Floor Disorders, can you identify any barriers to screening?

**Post-Intervention Survey: Provider Acceptability/Feasibility Questionnaire**

Email Address (fill in blank)

Provider Designation (drop-down): OB/GYN, CNM, NP, Resident (PGY-1, PGY-2, PGY-3, PGY-4)

Years OB experience: 0-2, 2-5, 5-10, 10-15, 15-20, 20+

1) Using the Cozean screening tool increased appointment duration time.

Strongly Disagree	Disagree	Neutral	Agree	Strongly Agree

2) This tool directed provider-patient conversation.

Strongly Disagree	Disagree	Neutral	Agree	Strongly Agree

3) The screening tool assisted with identifying patients warranting referral to Pelvic Floor Physical Therapy.

Strongly Disagree	Disagree	Neutral	Agree	Strongly Agree

4) This screening tool was helpful.

Strongly Disagree	Disagree	Neutral	Agree	Strongly Agree

5) I will continue using this screening tool.

Strongly Disagree	Disagree	Neutral	Agree	Strongly Agree

6) I will recommend this screening tool to other providers.

Strongly Disagree	Disagree	Neutral	Agree	Strongly Agree

7) It is feasible to integrate Pelvic Floor Disorder screening into my practice.

Strongly Disagree	Disagree	Neutral	Agree	Strongly Agree

8) I am satisfied with the postpartum Pelvic Floor Disorder screening process using the Cozean screening tool.

Strongly Disagree	Disagree	Neutral	Agree	Strongly Agree

9) Addressing Pelvic Floor Disorders in the early postpartum period improves women’s quality of life and military career.

Strongly Disagree	Disagree	Neutral	Agree	Strongly Agree

10) Any further comments about the project you would like to share? ­­
